# The governance of surgical innovation in the UK National Health Service

**DOI:** 10.1093/bjs/znag024

**Published:** 2026-03-13

**Authors:** Jane M Blazeby, Hollie S Richards, Sian Cousins, Lucy Wallis, Adrian Clarke, Stuart Metcalfe, Wendy Frost, Sarah Waters, Sanjoy Shah, Joydeep Grover, James Byrne, Diana Ward, Richard Dacombe, Louisa Wickham, Matthew D Gardiner, Bethany Bal, Claire Steel, Susan Pywell, Manal Etemadi, Jonathan Ives, Richard Huxtable, Kerry N L Avery, Leila Rooshenas, Daisy Elliott

**Affiliations:** NIHR Bristol Biomedical Research Centre, University of Bristol and University Hospitals Bristol and Weston NHS Foundation Trust, Bristol, UK; NIHR Bristol Biomedical Research Centre, University of Bristol and University Hospitals Bristol and Weston NHS Foundation Trust, Bristol, UK; NIHR Bristol Biomedical Research Centre, University of Bristol and University Hospitals Bristol and Weston NHS Foundation Trust, Bristol, UK; NIHR Bristol Biomedical Research Centre, University of Bristol and University Hospitals Bristol and Weston NHS Foundation Trust, Bristol, UK; University Hospitals Bristol and Weston NHS Foundation Trust, Bristol, UK; University Hospitals Bristol and Weston NHS Foundation Trust, Bristol, UK; North Bristol NHS Trust, Bristol, UK; North Bristol NHS Trust, Bristol, UK; North Bristol NHS Trust, Bristol, UK; North Bristol NHS Trust, Bristol, UK; University Hospitals Southampton NHS Foundation Trust, Southampton, UK; University Hospitals Southampton NHS Foundation Trust, Southampton, UK; University Hospitals Southampton NHS Foundation Trust, Southampton, UK; Moorfields Eye Hospital NHS Foundation Trust, London, UK; Frimley Health NHS Foundation Trust, Frimley, UK; Frimley Health NHS Foundation Trust, Frimley, UK; Frimley Health NHS Foundation Trust, Frimley, UK; NIHR Bristol Biomedical Research Centre, University of Bristol and University Hospitals Bristol and Weston NHS Foundation Trust, Bristol, UK; NIHR Bristol Biomedical Research Centre, University of Bristol and University Hospitals Bristol and Weston NHS Foundation Trust, Bristol, UK; NIHR Bristol Biomedical Research Centre, University of Bristol and University Hospitals Bristol and Weston NHS Foundation Trust, Bristol, UK; NIHR Bristol Biomedical Research Centre, University of Bristol and University Hospitals Bristol and Weston NHS Foundation Trust, Bristol, UK; NIHR Bristol Biomedical Research Centre, University of Bristol and University Hospitals Bristol and Weston NHS Foundation Trust, Bristol, UK; NIHR Bristol Biomedical Research Centre, University of Bristol and University Hospitals Bristol and Weston NHS Foundation Trust, Bristol, UK; NIHR Bristol Biomedical Research Centre, University of Bristol and University Hospitals Bristol and Weston NHS Foundation Trust, Bristol, UK

*Dear Editor*,

Innovation is pivotal to improve healthcare. Although widely endorsed by healthcare providers, it inherently carries risk and patients may be harmed. An independent enquiry highlighted where this can cause patient harm and recommended better governance of innovation, emphasizing the need for systematic outcome monitoring, and transparent informed consent^1^. How this is achieved in the UK National Health Service (NHS) for surgery is complex. Whereas the introduction of drugs predominantly occurs within a research framework, the governance of surgical innovation is unclear. The National Institute of Health and Care Excellence (NICE) provides governance guidance for about 30 new interventional procedures annually. NICE may recommend delivery within standard practice, with evidence generation (that is outcome data collected and shared), with research oversight, or advise that it should not be used^2^.

Governance guidance is also provided by NHS hospitals. Dedicated committees, known as New Procedures Committees (NPCs), consider applications for new intervention delivery within a single hospital. Committees usually consist of clinicians (for example surgeons, anaesthetists), administrative and clinical governance staff (for example managers with audit expertise). Unlike NICE or Health Research Authority (HRA) committees they do not usually include methodologists or patient members^3^. NPCs make similar recommendations to those made by NICE with decisions informed by national governance guidance, published evidence, and clinical judgement. When NICE guidance is unavailable NPCs/innovators are advised to notify NICE of the innovation. The HRA also provides governance for studies evaluating innovative procedures within a research framework^4^. Literature analyses shows that a minority of surgical innovations are introduced within research^5–7^. Local hospital NPCs are central to the governance of surgical innovation, although little is known about their workload and decision-making. This study explored the volume and type of governance decisions made by NPCs, and adherence to national governance guidance.

We report a 12-month service evaluation study (February 2022 to January 2023) that was undertaken in five English NHS hospitals (*Supplement Table S1*). An NPC was defined as a system appointed by the medical director to oversee the introduction of new procedures, including where this function operated alone or within a wider remit (for example clinical effectiveness group). Medical directors circulated a standardized email describing the study to clinicians before it began.

Eligible were innovative invasive interventions defined as those without evidence of safety and/or efficacy and those being used for the first time in the hospital^8^. Data extraction forms were developed, piloted and managed using REDCap (Research Electronic Data Capture) electronic tools. Numbers of committee meetings and new procedures were recorded. NPC decisions were noted and categorized by whether this followed national governance guidance. Where no guidance existed, it was recorded whether NICE was notified. The central research team independently examined all the guidance available and held meetings with participating hospitals to discuss the findings. A patient and public involvement and engagement group inputted into the study (details in *Supplement*).

In total, 35 procedures were discussed across 30 meetings with three being considered in more than one trust (*Supplement Table S1*). National guidance existed for 16 and was followed for 10 (*Supplement Table S2*). For the remaining six, NPCs recommended mostly lighter touch governance than NICE (that is standard rather than evidence generation/research). Of 22 new procedures without national governance guidance, 18 were approved for standard care with audit, three recommended for research, and one declined (*Table 1*). Two were notified to NICE and NICE recommended delivery within standard care (each considered a modification of an intervention with a known safety profile).

**Table 1:** Procedures (*n* = 22) considered in NPCs without available national governance guidance, whether NICE was notified and local governance decision

Brief description of new procedure/device	Type of new procedure	NICE notification	Local hospital decision
Device for ablating preinvasive malignant cells	New indication for use	Yes	Standard arrangements
Device for endoscopic suturing	First use in this hospital	Yes	Standard arrangements
Artificial implant graft used in a different place	New indication for use	No	Standard arrangements
New fixation technique	Modification	No	Standard arrangements
Replacing nitrous oxide with carbon dioxide for procedure	Modification	No	Standard arrangements
New device and enhanced delivery system	Modification	No	Standard arrangements
Convergent two-stage hybrid atrial fibrillation ablation	Modification	No	Standard arrangements
Navigational bronchoscopy for diagnosis of lung lesions	Novel device	No	Standard arrangements
Transbronchial cryobiopsy for interstitial lung disease	Novel device	No	Standard arrangements
Sentinel lymph node assessment in endometrial cancer	Modification	No	Standard arrangements
New drainage device	Novel device	No	Research only
Hypotony treatment for low ocular pressure visual loss	Novel procedure	No	Research only (10 patients approved first)
New laser device for glaucoma	Novel device	No	Advised not to use
Robotic-assisted benign gynaecological procedures	Modification	No	Standard arrangements^†^
Robotic-assisted malignant gynaecological procedures	Modification	No	Standard arrangements^†^
Robotic-assisted colorectal procedures	Modification	No	Standard arrangements^†^
Microrobot for image-guided intervention	Novel device	No	Standard arrangements
Ultrasound-guided percutaneous release of trigger finger	New procedure	No	Standard arrangements^‡^
Cyanoacrylate glue for hernia mesh fixation	Modification	No	Standard arrangements, NICE MIB 301 (2022)
*New device for anterior urethral strictures	New device	No	Research only, NICE MIB 241 (2021)
*New device for anterior urethral strictures	New device	No	Standard arrangements, NICE MIB 241 (2021)
A new system for managing intestinal failure	New device	No	Standard arrangements, NICE MIB 286 (2022)

^*^Device was considered by two hospitals. †NICE HTE21 (2025) approved five robotic technologies for evidence generation (special arrangements until 2024). ‡Academy of Royal Medical Colleges 2024 (AOMRC) recommended standard arrangements, MIB = Medtech Innovation Briefings (no longer produced after April 2023).

Hospitals sometimes reported identifying national guidance for these procedures, although when reviewed by the research team this was not always the case. For example, Medtech Innovation Briefings that do not make governance recommendations were cited (*n* = 3). Three hospitals approved robotic-assisted surgery for use in standard care citing NICE guidance for laparoscopic surgery (*Table 1*).

This study explored the governance of surgical innovation in a sample of five NHS hospitals. Hospitals made governance decisions largely independently, that is without notifying NICE, following national guidance or referring to research. This practice is of concern. It may jeopardize incremental safety assessment of new procedures and informed patient consent. *Figure 1* 'Algorithm for selecting NHS governance for new interventional procedures' illustrates how governance for innovative procedures could be strengthened in the NHS. It recommends that NPCs follow national guidance when available, and, for procedures without NICE guidance, that NICE be notified. It is recommended that NICE’s governance responses to notifications are shared across the NHS to provide equitable advice to all hospitals and that an open national database of new procedures being undertaken in hospitals is established.

**Fig. 1.**
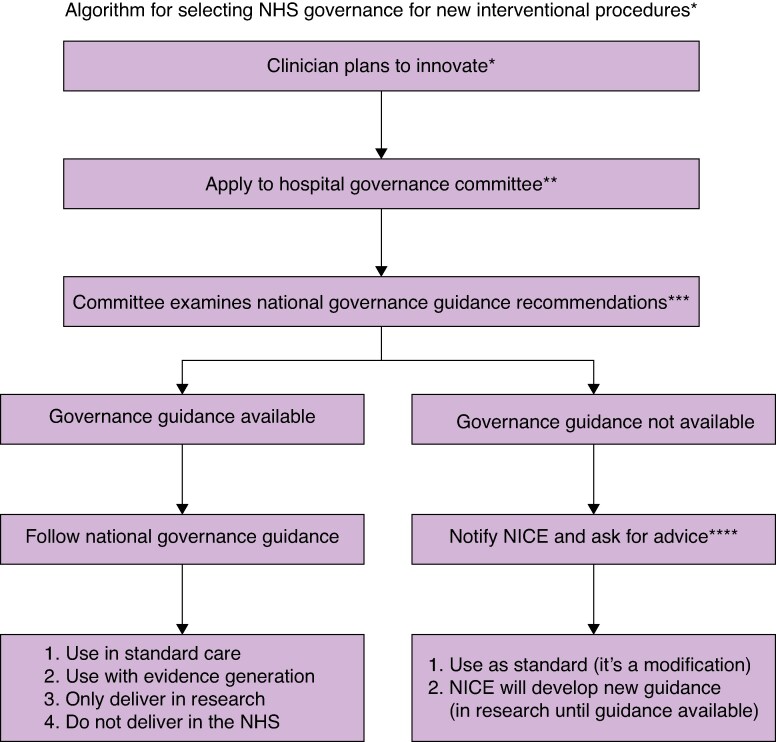
Algorithm for selecting NHS governance for new interventional procedures **A procedure is considered innovative if it does not have published safety and efficacy data.^8^ **In the NHS it the responsibility of the medical director of each hospital to ensure governance of new invasive interventional procedures is appropriate. This is often delegated to committees such as, e.g. ‘New Procedure Committees', ‘Clinical Effectiveness Committees’ etc.^2^ ***NICE = The National Institute of Health and Care Excellence provide governance guidance for 30 new invasive interventional procedures annually. **** NICE is notified via this link, https://tinyurl.com/y7x79h7r (accessed 4 February 2026).

The UK governance of surgical innovation is similar to that of other high-income countries^9,10^. Hospitals provide independent governance guidance for delivery of new procedures without a nationally agreed approach, and a minority are delivered within research. One study conducted in England and Wales analysed hospital governance policies and found variation in the type of invasive procedures for delivery with local approval, limited research guidance and deficiencies with patient information provision and informed consent^3,11,12^. Guidance for reporting and monitoring adverse events and when to abandon a procedure was lacking^12^.

Although this study is original and prospective in design, it had limitations. Data about the time NPCs took to make governance decisions were not captured. Obtaining local governance approvals for innovation delivery may be quicker and less onerous than using other mechanisms, although this requires further investigation. Another limitation is that participating hospitals were aware that they were being monitored and that may have influenced decision-making. Despite this, compliance with NICE guidance was very low. Additionally, the sample was small and practices may differ across the UK.

This study highlights how existing systems for the oversight of surgical innovation are disconnected. Although NICE provides governance guidance, it is not always followed, and a significant proportion of new procedures are being undertaken without notification to NICE or with research oversight. There is a need for better communication and agreement among hospital NPCs, NICE, and the HRA for the governance of innovation of invasive procedures.

## Supplementary Material

znag024_Supplementary_Data

## Data Availability

The study data are available from the corresponding author.
