# Supplementary material for: The governance of surgical innovation in the UK National Health Service
Source: Br J Surg. 2026 Mar 13;113(4):znag024. doi: 10.1093/bjs/znag024 (PMC13080363; doi:10.1093/bjs/znag024)
Supplement: znag024_Supplementary_Data [file znag024_supplementary_data.docx]

**The Governance of Surgical Innovation in the UK National Health Service**

JM Blazeby^1^, HS Richards,^1^ S Cousins^1^, L Wallis^1^, A Clarke^2^, S Metcalfe^2^, W Frost^3^, S Waters^3^, S Shah^3^, J Grover^3^, J Byrne^4^, D Ward^4^, R Dacombe^4^, L Wickham^5^, M D. Gardiner^6^, B Bal^6^, C Steel^6^, S Pywell^7^, M Etemadi^1^, J Ives^1^, R Huxtable^1^, KNL Avery^1^, L Rooshenas^1^ and D Elliott^1.^

^1^NIHR Bristol Biomedical Research Centre, University of Bristol and University Hospitals Bristol and Weston NHS Foundation Trust,

^2^University Hospitals Bristol and Weston NHS Foundation Trust,

^3^North Bristol NHS Trust,

^4^University Hospitals Southampton NHS Foundation Trust,

^5^Moorfields Eye Hospital NHS Foundation Trust,

^6^Frimley Health NHS Foundation Trust

**Corresponding author:** Professor Jane M Blazeby, NIHR Bristol Biomedical Research Centre, University of Bristol and University Hospitals Bristol and Weston NHS Foundation Trust, email: j.m.blazeby@bris.ac.uk.

ORCID ID: 0000-0002-3354-3330

X @BlazebyJ

**Acknowledgements**

The authors would like to thank the Medical Directors and administrative teams at each of the trusts involved in this project.

**Author Contributions**

JMB conceived the study and obtained funding. Methodology was developed by JMB, SC, HSR, AC, SM, JB, LW, MDG, JI, SP and RH. Data curation and project administration was undertaken by SC, LW, WF, SW, DW, RD, and CS. Formal analysis was undertaken by SC, LW, JMB, AC, SM, SS, JG, JB, LW, MDG and BB. The original draft was written by HSR and JMB. Writing review and editing was undertaken by HSR, JMB, DE, SC, LW, AC, SM, WF, SW, SS, JG, JB, DW, RD, LW, MDG, BB, CS, SP, JI, KA, ME and RH. All authors reviewed the article

**Supplementary Materials - Index**

| **Supplementary Methods** |  |
| --- | --- |
| PPIE statement | *page 3* |
| Governance statement | *page 3* |
| **Supplementary Figures and Tables** |  |
| Table 1 Number of new procedure meetings.  Table 2. New procedures (n=16) with nationally available governance guidance or guidance under development and local hospital governance decision | *page 4*  *page 5* |
| **References** | *page 7* |
|  |  |

**Supplementary Methods**

*Patient and public involvement and engagement*

A patient and public involvement and engagement (PPIE) group was established at the study outset. This included two members, one of whom had first-hand experience of the significant harms caused by innovative use of mesh. The group met regularly with a member of the research team (SC) and one PPIE member attended study team meetings. The PPIE group and representation provided input into study design, conduct, analyses and reporting.

*Study governance*

This study was registered in each participating trust as a prospective service evaluation.

**Supplementary Figures and Tables**

| **Trust details**~ | **Meetings**  **n=30** | **Applications**  **n=38**** |
| --- | --- | --- |
| South West England, teaching, foundation | 7 | 6 |
| South West England, large, non-foundation | 6 | 1 |
| South East England, teaching, foundation | 11 | 19 |
| London, specialist, foundation | 4 | 7 |
| South East England, large, foundation* | 2 | 5 |

**Table 1 Number of new procedure meetings and eligible applications by type of NHS Trust.**

*This Trust enrolled from September 2022 – January 2023 only.

**3 procedures were considered in 3 trusts, total number of new procedures n=35.

~ Area in England, teaching, large or specialist and Foundation/Non-Foundation(1)

| **Brief description of new procedure/device** | **Type of new procedure** | **Guidance, year of publication & recommendation** | **Local hospital decision** |
| --- | --- | --- | --- |
| *Artificial joint replacement for end-stage (osteoarthritis)* | New device | NICE IPG 111 (2005), standard arrangements | Standard arrangements |
| *Artificial joint replacement for end-stage (osteoarthritis)* | New device | NICE IPG 111 (2005), standard arrangements | Standard arrangements |
| Selective internal radiation therapy for primary hepatocellular carcinoma | New procedure & radiation therapy | NICE IPG 460 (2013), standard arrangements | Standard arrangements |
| Sacral nerve stimulation for faecal incontinence | Nerve stimulation | NICE IPG 99 (2004), standard arrangements | Standard arrangements |
| *New technology for lower urinary tract symptoms secondary to benign prostatic hyperplasia | New procedure | Medical Technologies Guidance 49 (2020), standard arrangements | Standard arrangements |
| *New technology for lower urinary tract symptoms secondary to benign prostatic hyperplasia | New procedure | Medical Technologies Guidance 49 (2020), standard arrangements | Standard arrangements |
| Pulsed field ablation for atrial fibrillation | Modification | NICE IPG 806 (2025), standard arrangements. | Standard arrangements |
| Transcervical ultrasound-guided radiofrequency ablation for symptomatic uterine fibroids | Surgery & radiofrequency ablation | NICE IPG 689 (2012), special arrangements | Standard arrangements |
| Aortic remodelling hybrid stent insertion during surgical repair of an acute type A aortic dissection | New device | NICE IPG 733 (2022), special arrangements | Standard arrangements |
| Percutaneous mechanical thrombectomy for acute leg deep vein thrombosis | New device & procedure | NICE IPG 651 (2019), special arrangements | Standard arrangements |
| Biodegradable spacer to use during radiotherapy | New device | NICE IPG 752 (2023), special arrangements. | Standard arrangements |
| Permanent his-bundle pacemaker implantation for treating heart failure | New procedure | NICE IPG 694 (2012), research only | Standard arrangements |
| Device open-angle glaucoma | New procedure & device | NICE IPG 745 (2022), special arrangements | Research only |
| **Transperineal laser ablation of the prostate | New device | NICE DG 54 (2023), in development at time of study. When published recommendations varied by type of device | Advised not to use |
| Cryotherapy for chronic rhinitis | New procedure | NICE IPG 771 (2023), in development at time of study. When published it recommended research only. | Advised not to use |
| Endoscopic ultrasound-guided gallbladder drainage for acute cholecystitis when surgery is not an option | New procedure | NICE IPG 764 (2023) in development at time of study. When published it recommended standard arrangements. | Advised not to use |

NICE = National Institute of Clinical Excellence, IPG= Interventional Procedures Guidance, DG = Diagnostic Guidance,

*Procedure assessed by two trusts independently, **NICE notified unnecessarily.

**Table 2. New procedures (n=16) with nationally available governance guidance or guidance under development and local hospital governance decision**

**References**

1. Digital N. Estates Returns Information Collection Summary page and dataset for ERIC 2018/19: NHS Estates Returns Information Collection; 2019 [Available from: <https://digital.nhs.uk/data-and-information/publications/statistical/estates-returns-information-collection/england-2018-19>.
